# Enhancing blockchain technology adoption in governmental operations: A comprehensive framework for user adoption

**DOI:** 10.1371/journal.pone.0352781

**Published:** 2026-07-06

**Authors:** Aloka Gnanasekara, Anuradha Jayakody, Kasun Perera

**Affiliations:** 1 Faculty of Graduate Studies, Sri Lanka Institute of Information Technology, Malabe Province - Western Province, Sri Lanka; 2 Department of Computer Systems Engineering, Sri Lanka Institute of Information Technology, Sri Lanka; 3 Department of Finance, University of Colombo, Sri Lanka; Beijing Technology and Business University, CHINA

## Abstract

The purpose of this study is to investigate the factors that affect Blockchain adoption in governmental operations in Sri Lanka and to propose a comprehensive adoption framework for Blockchain technology in the Sri Lankan governmental operations. The Technology- Organization-Environment (TOE) framework is utilized due to its capacity to capture the complexities of technological adoption in the public sector, addressing both internal (organizational) and external (environmental) factors that influence the adoption process. Given the structural, regulatory, and data sensitivity challenges of governmental settings, the TOE framework integrates employee insights from technological, organizational, and environmental perspectives, making it adaptable to the public sector’s needs and scalable across various government entities. It also reflects the regulatory and operational requirements specific to the Sri Lankan public institutions, including essential compliance areas such as data privacy, security regulations, and government workflows, thereby offering a practical pathway for Blockchain adoption within the local context. This study employed statistical methods to ensure the validity and reliability of data collected through a structured questionnaire distributed to Grade I–IT Directors to capture their perceptions and experiences with Blockchain technology. Using the structural equation modelling (SEM), the study finds that all the technological, organizational, and environmental dimensions of the TOE framework are significantly associated with intention to adopt Blockchain technology in the Sri Lankan governmental operations. At a more specific level, trust, compatibility, security, higher authority support, monetary resources, rivalry pressure, and regulatory support were identified as significant predictors of adoption intention, while relative advantage, IT resources, and business partner pressure were not statistically significant, and firm size showed only weak support. These findings provide an empirically grounded framework for understanding Blockchain adoption intention in the Sri Lankan public sector and offer implications for future policy and implementation planning.

## 1. Introduction

The dawn of Blockchain technology has revolutionized digital transactions by enhancing transparency, security, and efficiency. Originally introduced as the foundational technology for Bitcoin by Nakamoto in 2008, Blockchain has evolved far beyond cryptocurrencies into applications spanning finance, healthcare, supply-chain management, and government operations [1]. It is a decentralized digital ledger that records transactions across multiple computers, guaranteeing immutability, cryptographic security, and transparency. Because its core principles, namely, decentralization, cryptography, and immutability, eliminate reliance on a single trusted authority, Blockchain is particularly suitable for environments where accountability and data integrity are essential [2].

Unlike traditional centralized systems, Blockchain operates on a peer-to-peer network that removes intermediaries [3]. Each participant maintains a synchronized copy of the ledger, reducing single-point failures and fostering trust. Once a transaction is recorded, it cannot be altered, ensuring permanent data integrity [4]. Public Blockchains offer full visibility, while private or permissioned networks still achieve greater transparency than conventional databases [5]. Cryptographic techniques further secure data and restrict unauthorized access, an advantage particularly valuable in sectors demanding high data integrity [6].

Across industries, Blockchain has proven transformative. In finance, it streamlines secure transactions and lowers intermediary costs; in healthcare, it strengthens data sharing and privacy [7]. Governments worldwide are now exploring Blockchain for transparent voting systems and secure public-record management [7]. The technology’s potential to improve the transparency, data security, process efficiency, and corruption reduction makes it especially relevant to public sector modernization.

Transparency is one of Blockchain’s most compelling benefits. By maintaining a decentralized, verifiable ledger, governments can enable citizens to track transactions, budgets, and allocations in real time [1,3]. Such openness builds public trust and deters fraud, especially in land registration and procurement [4]. Blockchain’s encryption safeguards sensitive data, protecting citizen information, legal records, and official communications [5,6]. Smart contracts, self-executing programs triggered by predefined conditions, can automate bureaucratic procedures, accelerating processes and reducing human error [7]. In areas like welfare disbursement, they ensure benefits are delivered accurately and promptly [8]. The immutable, auditable nature of Blockchain records makes corruption and data tampering extremely difficult [9,10].

Global experiences further demonstrate Blockchain’s public sector impact. Estonia and Dubai have integrated Blockchain into national registries, visa systems, and document management, resulting in cost reductions and improved efficiency [11]. For developing economies, Blockchain provides an opportunity to bypass legacy inefficiencies and introduce transparent, technology-driven governance [12].

Sri Lanka’s public administration faces long-standing inefficiencies, limited transparency, and data vulnerabilities. Bureaucratic delays, redundant procedures, and fragmented systems increase costs and reduce service quality [1,3]. Limited access to information fosters distrust, and opaque procurement practices have enabled corruption [4,5]. Cybersecurity risks also threaten the confidentiality of government and citizen data [6,7]. Blockchain offers a strategic response by enabling tamper-proof, verifiable records and secure data exchange among agencies [8,9,10].

Interest in Blockchain within Sri Lanka’s public sector is growing, but adoption remains nascent [11]. Major barriers include inadequate ICT infrastructure, ambiguous regulation, and insufficient organizational readiness [12–14]. Although digital infrastructure has improved, rural connectivity and reliable electricity remain concerns [13]. Moreover, effective implementation demands skilled personnel and clear policies governing data privacy, cybersecurity, and the legal status of Blockchain transactions [12,14]. The Technology–Organization–Environment (TOE) framework provides a comprehensive foundation for analyzing these challenges [15]. It allows simultaneous consideration of technological readiness, organizational capacity, and environmental pressures, enabling a holistic understanding of Blockchain adoption in government.

Blockchain’s transformative potential has motivated this research, which aims to examine how it can enhance Sri Lanka’s governance, transparency, and efficiency. Globally, Blockchain has improved service delivery and reduced corruption [16,17]. In Sri Lanka, persistent inefficiencies and public distrust underscore the urgency of such transformation. The research problem centers on identifying the technological, organizational, and environmental factors influencing Blockchain adoption in governmental operations. Trust remains a cornerstone of effective governance, yet perceptions of corruption and favoritism continue to erode public confidence [10,11]. Complex bureaucratic procedures delay services, inflate costs, and discourage citizen engagement [12,13]. Meanwhile, weak cybersecurity measures expose sensitive data to breaches and manipulation, threatening both privacy and institutional credibility [14,15,18]. Although Sri Lanka is pursuing digital government reforms, Blockchain adoption lags due to infrastructural gaps, limited organizational preparedness, and regulatory uncertainty [19].

Internationally, countries such as Estonia and Dubai have demonstrated how Blockchain can enhance transparency, efficiency, and citizen participation [20]. Inspired by these successes, the present study attempts to bridge the gap between Blockchain’s technological potential and its practical implementation in Sri Lanka’s public sector. Understanding how technological, organizational, and environmental factors jointly determine adoption readiness provides the basis for a structured framework to guide national-level integration.

Blockchain could fundamentally reshape administrative processes by enhancing transparency, automating workflows, and ensuring data integrity. Technologically, it provides verifiable, tamper-resistant records that reinforce trust and reduce opportunities for corruption. Organizationally, leadership commitment, sufficient funding, and skilled personnel are critical for successful implementation [21,22]. Environmentally, effective regulation, inter-agency collaboration, and competitive pressure can accelerate adoption [23,24]. Together, these factors influence how government institutions assess, pilot, and institutionalize Blockchain solutions.

The theoretical contribution of this study is mainly four-fold. First, while extensive research has examined Blockchain in private-sector domains such as operations, supply chains, and accounting [25]–[27], public-sector adoption in developing countries has received more limited attention [28]. In a related study, [29] develop a Blockchain readiness index and an importance-performance map to evaluate macro-level organizational readiness parameters and data-driven analytics for Blockchain adoption across Sri Lankan public sector organizations. In contrast, to address institutional specificity, we developed a comprehensive questionnaire following [30] to collect the required data by specifically targeting the Sri Lankan government institutions officially mandated by government gazette notification (See [href:https://pubad.gov.lk/web/images/latest_document/service_minutes/2014/1431938178-1894-26--e-.pdf]https://pubad.gov.lk/web/images/latest_document/service_minutes/2014/1431938178-1894-26--e-.pdf) to maintain dedicated IT departments overseen by Grade I IT Directors. These key informants, the gazetted Grade I IT Directors, serve as the primary operational gatekeepers responsible for implementing state IT policies within their respective institutions. Beyond this theoretical contribution, we translate our findings into a structured and practical Blockchain adoption framework and Blockchain adoption checklist to support implementation planning in governmental operations, thereby further differentiating our study from [29].

Second, the study refines the application of the TOE framework for public administration by showing that Blockchain adoption in government is not merely a question of technological usefulness, but is conditioned by legacy-system compatibility, leadership commitment, financial and technical readiness, and legal clarity [32,35,36,37,38–41]. Third, the study contributes empirically by identifying which TOE sub-dimensions are most salient in this context, thereby showing that adoption intention in the Sri Lankan governmental operations depends more on institutional fit, governance support, and regulatory legitimacy than on technological attractiveness alone. Fourth, beyond its theoretical contribution, the study translates these findings into a structured Blockchain Adoption Framework and Implementation Checklist to guide implementation planning in governmental operations.

More broadly, although prior studies have examined Blockchain applications in government, the theoretical increment of the present study lies in moving beyond general application-oriented discussion toward a context-specific explanation of adoption in public administration [6,30], and [31]. In particular, this study argues that Blockchain adoption in governmental operations should be understood as an institutionally embedded organizational process shaped by the interaction of technological compatibility, leadership support, resource readiness, and regulatory conditions. This is especially important in developing-country contexts, where infrastructure gaps, limited trained personnel, and regulatory uncertainty may alter the relative importance of adoption drivers [19,42]. Accordingly, this study does not merely apply the TOE framework to a new setting; it refines the TOE lens for the public sector by showing how technological, organizational, and environmental dimensions operate together under the structural realities of the Sri Lankan governmental institutions.

The rest of the paper is structured as follows. Section two elaborates the related literature, while the third section explains the methods and materials used in the study. Section four discusses the findings of this study and section five concludes the paper.

## 2. Literature review and hypotheses development

Blockchain technology has emerged in the twenty-first century as a transformative digital innovation, offering a decentralized and secure method for recording and managing data across industries. Originally developed as the foundation for Bitcoin, Blockchain has evolved beyond its financial roots to revolutionize multiple domains, including healthcare, supply chains, and government operations [2]. Its capacity to provide transparent, immutable, and tamper-proof transaction records enhances the accountability and efficiency of public sector processes [6]. In developing countries such as Sri Lanka, where corruption, inefficiency, and lack of transparency impede progress, Blockchain can establish a secure data environment that fosters trust, reduces administrative delays, and safeguards record integrity [19,21]. However, successful adoption requires evaluating technological readiness, organizational capabilities, and the broader regulatory and societal context [21]. The TOE framework provides a robust structure for examining these interrelated factors [15].

Since its introduction in 2008, Blockchain has expanded from cryptocurrency applications to a wide range of uses across sectors [2]. Its core features, decentralization, immutability, smart contracts, and consensus mechanisms, define its operational strength. Decentralization distributes data across nodes instead of relying on a central authority, thereby improving transparency and resilience [14,15]. Immutability ensures that once data are validated and recorded, they cannot be altered without network consensus, making it ideal for environments that demand reliable audit trails [16]. Smart contracts, which automatically execute transactions once predefined conditions are met, reduce manual intervention and accelerate processes [17]. Consensus mechanisms such as proof of work and proof of stake maintain trust and data consistency within the network by ensuring agreement among participants have driven Blockchain’s expansion into diverse sectors including finance, healthcare, and supply chain management [18].

In finance, Blockchain has disrupted traditional models by enabling fast and secure peer-to-peer transactions while minimizing intermediaries [19,20]. Healthcare systems use Blockchain to ensure secure, interoperable patient data exchange, improving accuracy and patient safety [21]. Additionally, Blockchain plays a crucial role in verifying the authenticity of pharmaceuticals throughout the supply chain [22]. In logistics, Blockchain records every transaction transparently, enabling real-time tracking, authenticity verification, and regulatory compliance [23,24]. Through these applications, Blockchain has consistently reduced fraud and operational inefficiencies while fostering transparency [28]. With integration into emerging technologies such as artificial intelligence (AI) and the Internet of Things (IoT), Blockchain continues to offer innovative possibilities for digital governance.

Public administrations worldwide have leveraged Blockchain to strengthen transparency, data security, and efficiency. Estonia, a pioneer in e-governance, uses Blockchain to secure national registries including land, health, and identity systems, alongside its e-residency program, which enables secure digital identities and cross-border business registration [20]. These initiatives have significantly improved operational efficiency and citizen trust. Similarly, the United Arab Emirates’ Emirates Blockchain Strategy 2021 aims to digitize all government documents by 2024. Projects such as the Dubai Land Department’s Blockchain-based property transfer system have reduced processing time and administrative costs while improving data integrity [6,30,31]. These international examples demonstrate that Blockchain adoption can reduce bureaucracy, standardize control points, and enhance service delivery, positioning it as a critical technology for effective governance [6].

The adoption of Blockchain technology in the public sector is influenced by a complex interplay of technological, organizational, and environmental factors. On the technological side, compatibility with existing systems facilitates smooth implementation, while incompatibility with legacy platforms can result in high costs and operational friction [32]. Perceived security is another determinant, as public-sector institutions must be assured of Blockchain’s cryptographic resilience and privacy controls [33]. Scalability, ensuring that Blockchain systems can handle large transaction volumes, is essential for high-demand public applications [34]. The organizational dimension encompasses leadership commitment, communication, and resource readiness. Leaders who advocate for technological change can mobilize resources, reduce resistance, and inspire staff engagement [35]. Meanwhile, adequate financial and technical resources determine project feasibility and sustainability [36]. The environmental dimension relates to regulatory clarity and policy support. Supportive regulations enhance adoption, whereas ambiguity or restrictive laws create uncertainty and slow implementation [37].

Despite its potential, Blockchain adoption faces several barriers. Many governments, particularly in developing countries, lack robust ICT infrastructure, such as high-speed internet, secure data centers, and cybersecurity systems [38]. Integration with existing legacy systems often poses significant challenges due to incompatibility and limited interoperability [39]. High costs of implementation, maintenance, and training remain deterrents for resource-constrained public institutions [39]. Furthermore, regulatory and legal challenges complicate adoption. Data protection laws, including the European Union’s GDPR, impose strict controls over data management, which can conflict with Blockchain’s immutable nature [40]. Accountability issues also arise, as decentralized systems make it difficult to assign liability for errors or breaches [41]. Hence, balancing technological advancement with governance and legal compliance is critical for sustainable adoption.

In Sri Lanka, Blockchain technology could address many entrenched public-sector challenges, including inefficiency, corruption, and data insecurity [42]. It can promote transparency by providing immutable and auditable records of transactions, thereby reinforcing public accountability [42–44]. Data decentralization reduces the risks associated with centralized storage, while cryptographic –validation ensures data authenticity and security [18]. Blockchain can streamline administrative processes by automating verification tasks and minimizing manual errors [19]. For instance, in land management, Blockchain-based registries could automate ownership verification and reduce disputes [17,21]. Similarly, in public procurement, Blockchain’s transparency could prevent corruption and enhance the integrity of government contracting [20]. However, Sri Lanka must overcome several challenges before realizing these benefits. Technological readiness remains uneven due to infrastructure gaps and inadequate digital literacy [20]. Additionally, the lack of a clear regulatory framework and trained personnel constrains large-scale implementation [19,42]. Institutional change management and policy reform are therefore vital prerequisites for effective adoption.

Several theoretical models explain the process of technology adoption in the public sector. The Unified Theory of Acceptance and Use of Technology (UTAUT) emphasizes user perceptions such as performance expectancy and social influence [45,46]. The Diffusion of Innovations (DOI) theory highlights factors like relative advantage, compatibility, and observability [47], while the institutional theory focuses on normative and coercive pressures within bureaucratic contexts [48]. Although these frameworks provide valuable insights, they often fail to account for the multi-layered institutional and environmental complexities of government technology adoption. The TOE framework overcomes this limitation by integrating all three dimensions, technological, organizational, and environmental, into a comprehensive model [49–52]. It allows for an assessment of both internal and external determinants of adoption, making it particularly relevant for evaluating Blockchain adoption in the public sector [4,6,26].

Accordingly, under the technological dimension, factors such as relative advantage, trust, compatibility, and security are expected to play significant roles in influencing Blockchain adoption, while Blockchain technology enhances operational efficiency, transparency, and data security, contributing to improved management of government resources and services [26]. Moreover, the aspects such as higher authority support, firm size, monetary resources, and IT resources are critical under the organizational dimension where the top management commitment and sufficient resource allocation are vital for effective Blockchain integration [26,38]. Further, under the environmental dimension, the competitive pressures and supportive regulations create an enabling environment that motivates institutions to embrace Blockchain [53].

Therefore, based on the foregone literature, three research hypotheses were formulated to examine the Blockchain adoption in the Sri Lankan governmental operations as follows.

**Hypothesis 1:** Technological factors positively influence the intention of adopting Blockchain technology in the Sri Lankan governmental operations.

**Hypothesis 2:** Organizational factors positively influence the intention of adopting Blockchain technology in the Sri Lankan governmental operations.

**Hypothesis 3:** Environmental factors positively influence the intention of adopting Blockchain technology in the Sri Lankan governmental operations.

## 3. Materials and methods

Following prior studies [30], an online survey technique was employed to collect the required data. The study adopted a key-informant sampling approach and targeted executive-level officers in the Sri Lanka Information and Communication Technology Service within the public sector. The Gazette classifies this service as part of the executive public-service category and defines its role as using information and communication technology to build an excellent public service and implement government ICT policies while maintaining coordination with internal and external institutions (see [href:https://pubad.gov.lk/web/images/latest_document/service_minutes/2014/1431938178-1894-26--e-.pdf]https://pubad.gov.lk/web/images/latest_document/service_minutes/2014/1431938178-1894-26--e-.pdf). The role profile of the service includes drafting ICT policies, identifying institutional ICT solutions, conducting technical evaluation, implementing and evaluating ICT projects, generating management information, and coordinating with internal and external institutions. Accordingly, the sampling frame consisted of Grade I ICT Directors and equivalent senior ICT officers serving across government ministries and departments. This choice was made because these officers are not only responsible for technical administration, but also for identifying institutional ICT solutions, coordinating implementation, supporting policy execution, and aligning digital initiatives with public-sector procedures and administrative requirements. Therefore, they were considered appropriate respondents for assessing organizational readiness for Blockchain adoption in governmental operations.

Moreover, the sample also covered a diverse range of institutional types and organizational sizes across the Sri Lankan public sector. It includes public sector organizations such as the President’s Office, Office of the Cabinet of Ministers, and Ministry of Education, Ministry of Foreign Affairs, Ministry of Finance as well as large service departments and operational agencies such as the Ministry of Health, Department of Examinations, Department of Railways, Department of Census and Statistics, Department of Inland Revenue, Department of Pensions, and the Department of Information Technology Management. These institutions differ substantially in mandate, service scope, and internal scale. Therefore, the final sample was not limited to one type of governmental body; rather, it represented policy-making institutions, regulatory and administrative departments, citizen-service agencies, revenue-related entities, and technical/data agencies. This diversity improves the representativeness of the study for public-sector Blockchain adoption by covering agencies of different functions and sizes.

To further illustrate the breadth of the sampling frame, an institutional distribution table is provided in the S1 Appendix, showing the range of ministries, departments, and central government institutions represented in the executive ICT cadre. Accordingly, invitations describing the study objectives and requesting voluntary participation were sent to 150 identified officers based on official ministry and departmental sources. A pilot study was initially conducted with 30 respondents from this sampling frame to evaluate the clarity and contextual suitability of the questionnaire. After minor revisions, the final questionnaire was distributed to the full sample, from which 108 usable responses were received, yielding a response rate of 72%**,** which is acceptable for this type of research [54].

The required data was collected using a structured questionnaire (see S2 Appendix). The questionnaire development process involved four stages. First, the measurement items were identified following [30] to ensure data was collected using validated instruments to reflect that Blockchain adoption is within the TOE framework. Second, these items were then adapted to the context of Sri Lankan public sector organizations to improve contextual relevance without altering their original conceptual content. Third, the draft instrument was structured around the three TOE dimensions and measurement items for the corresponding latent variables together with the dependent construct of intention to adopt Blockchain technology. A five-point Likert scale ranging from “Strongly Disagree (1)” to “Strongly Agree (5)” was used to record responses. Fourth, a pilot study was conducted with 30 respondents from the target population to assess item clarity, contextual suitability, and response consistency. Based on the pilot feedback, minor wording revisions were made before final administration. This process helped strengthen the content validity and contextual appropriateness of the instrument. Details of all latent variables, measurement items, and source references are provided in the S4 Appendix.

In this study, data privacy and confidentiality were prioritized to protect participant anonymity and maintain the integrity of sensitive governmental information. The questionnaire distribution was administered via Google Forms over a period of three months from 22/05/2024–22/08/2024 after obtaining formal ethical approval from the Institutional Ethics Review Committee (IERC) of the Sri Lanka Institute of Information Technology (SLIIT) under the official approval reference number SLIIT-IERC-2024–0821.The questionnaire was designed without the collection of any personally identifiable information (PII), ensuring that participants’ identities could not be traced. It also clearly outlined that responses would be used solely for academic purposes, underscoring the study’s academic nature. Data collected was securely stored, and strict protocols were followed to prevent unauthorized access, preserving the confidentiality of the information related to governmental processes.

The informed consent process was designed to transparently inform participants about the study and their rights. All participants were adults employed in the Sri Lankan public sector institutions; therefore, parental or guardian consent was not required. This study did not involve any medical records, patient data, or other sensitive health information. Each participant received a description of the study’s purpose, the voluntary nature of participation, and assurances of response confidentiality. Participants were also informed of their right to withdraw from the study at any time without consequences. The consent statement clarified that no PII would be collected, reinforcing anonymity and protecting participants’ privacy. Through this process, the study maintained ethical standards and participant trust, while respecting their autonomy and data security.

**Fig 1:**
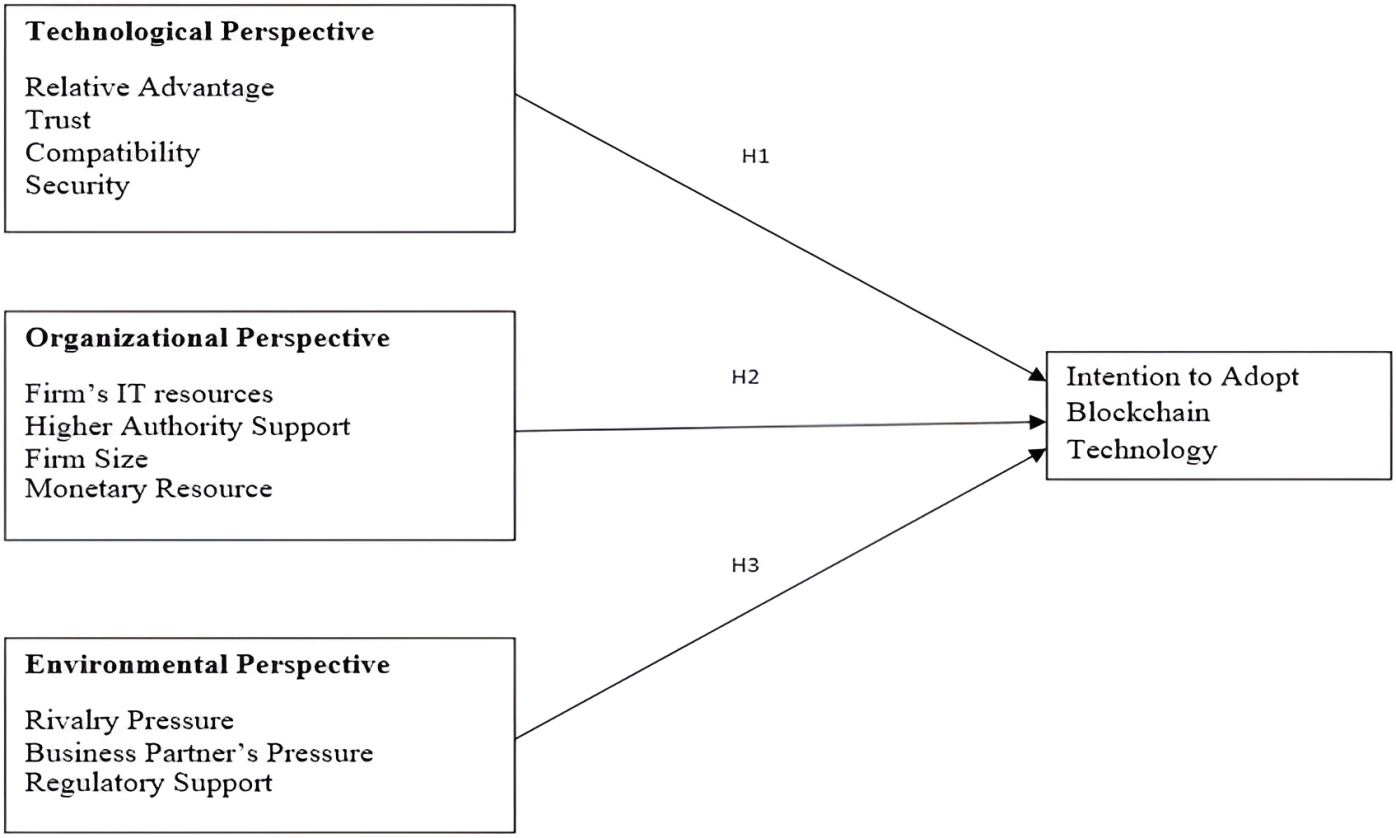
Conceptual framework.

The conceptual framework (see Fig 1) of the study was developed based on the TOE framework, integrating the three main dimensions that collectively influence Blockchain adoption within the Sri Lankan governmental operations. The model hypothesizes that these dimensions directly affect the intention to adopt Blockchain technology in the public sector. The technological dimension comprises relative advantage, trust, compatibility, and security. The organizational dimension includes higher authority support, IT resources, firm size, and monetary resources, while the environmental dimension involves rivalry pressure, business partner pressure, and regulatory support. Together, these factors represent the independent variables influencing the dependent variable, which is the intention to adopt Blockchain technology in public institutions. This conceptual model guides the empirical analysis of the study and reflects the hypotheses developed in the previous chapter.

To evaluate the quality of the measurement instrument, several procedures were applied to establish reliability and validity. First, internal consistency reliability was assessed using Cronbach’s alpha. A value above 0.70 is generally considered acceptable, indicating that the items consistently measure the same latent construct [55,56]. Second, construct validity was assessed through factor-analytic procedures. The Kaiser–Meyer–Olkin (KMO) measure and Bartlett’s test of sphericity were used to confirm sampling adequacy and the suitability of the correlation matrix for factor analysis [57]. Third, factor loadings were examined to determine whether the observed indicators loaded satisfactorily on their respective constructs. Items with acceptable loading values were retained for subsequent analysis. Together, these procedures ensured that the measurement model was sufficiently reliable and valid for hypotheses testing through Structural Equation Modeling (SEM).

To test the research hypotheses and examine relationships among variables, the study employed SEM using AMOS 26.0 software. While standard SEM provides robust validation of causal relationships, [58] integrated SEM with machine learning and explainable AI to predict Blockchain adoption readiness, highlighting the growing use of hybrid computational approaches in contemporary research. Nevertheless, to align with the objectives of the study, standard SEM was chosen because it allows simultaneous estimation of multiple interrelated dependence relationships among observed and latent variables. Two models were analyzed. The first model tested the direct effects of the three TOE dimensions on the intention to adopt Blockchain technology (IABT). The second model further decomposed each dimension into its sub-factors, such as relative advantage, trust, security, higher authority support, firm size, and regulatory support, to determine their specific effects on adoption intention. SEM’s integrated approach to confirmatory factor analysis and path modeling provides a comprehensive understanding of how individual factors and broader dimensions interact to influence adoption behavior. Model fit indices, including Chi-square/df, Goodness-of-Fit Index (GFI), Comparative Fit Index (CFI), and Root Mean Square Error of Approximation (RMSEA), were used to evaluate the fitness of the model to the data [59].

## 4. Results and discussion

To assess the internal consistency and validity of the constructs used in this study, reliability and validity tests were performed. As noted by [57], these tests determine the extent to which the indicators consistently measure their respective latent variables. Following [60], the reliability of each construct was evaluated using Cronbach’s alpha. The results of this test are presented in Table 1. All Cronbach’s alpha values were above 0.7, exceeding the recommended threshold of 0.70. Hence, it indicates a strong internal consistency. Therefore, the measurement items used in this study effectively captured the dimensions of the TOE framework. Table 1 shows the results of the Cronbach alpha analysis.

**Table 1:** Reliability test.

Construct	Items	Cronbach’s alpha
Relative Advantage	TRA1	0.922
	TRA2	
	TRA3	
	TRA4	
Trust	TTR1	0.855
	TTR2	
	TTR3	
Compatibility	TC1	0.919
	TC2	
	TC3	
	TC4	
Security	TSE1	0.911
	TSE2	
	TSE3	
	TSE4	
Higher Authority Support	OHAC1	0.947
	OHAC2	
	OHAC3	
	OHAC4	
	OHAC5	
Firm Size	OOFS1	0.916
	OOFS2	
	OOFS3	
Monetary Resources	OMR1	0.937
	OMR2	
	OMR3	
	OMR4	
	OMR5	
IT resources	OFR1	0.898
	OFR2	
	OFR3	
	OFR4	
Rivalry Pressure	ERP1	0.938
	ERP2	
	ERP3	
	ERP4	
Business Partner’s Pressure	EBP1	0.806
	EBP2	
	EBP3	
	EBP4	
	EBP5	
	EBP6	
Regulatory Support	ERS1	0.901
	ERS2	
	ERS3	
	ERS4	
Intention to Adopt Blockchain Technology	ITA1	0.896
	ITA2	
	ITA3	

**Note.** This table shows the results of the reliability test. All variables are defined in the S4 Appendix.

In addition, the Kaiser-Meyer-Olkin (KMO) measure of sampling adequacy and Bartlett’s test of sphericity were conducted to ensure that the data were suitable for factor analysis. The KMO value was 0.939, which is well above the recommended threshold of 0.60, indicating that the sample was appropriate for factor analysis [57]. Bartlett’s test statistic was also significant (p < 0.001), confirming that the correlation matrix was not an identity matrix and that the variables were sufficiently correlated for further analysis. Consequently, factor analysis was performed to identify the relationships among observed variables and their respective latent constructs. The results are presented in Table 2. All factor loadings exceeded 0.5, which meets the acceptance level suggested by [60] and aligns with the findings of previous studies [30]. Hence, these results indicate that the observed items adequately represented their intended latent constructs, providing evidence that the questionnaire possessed acceptable reliability and construct validity for the SEM analysis.

**Table 2:** Factor analysis.

Item	Factor loading	Item	Factor loading
TRA1	0.878	ERP4	0.891
TRA2	0.891	EBP1	0.842
TRA3	0.867	EBP2	0.864
TRA4	0.843	EBP3	0.848
TC1	0.837	EBP4	0.817
TC2	0.846	EBP5	0.896
TC3	0.847	ERS1	0.796
TC4	0.894	ERS2	0.880
OHAC1	0.847	ERS3	0.894
OHAC2	0.863	ERS4	0.894
OHAC3	0.912	TTR1	0.872
OHAC4	0.829	TTR2	0.841
OHAC5	0.877	TTR3	0.855
OOFS1	0.902	TSE1	0.852
OOFS2	0.888	TSE2	0.882
OOFS3	0.900	TSE3	0.882
OMR1	0.864	TSE4	0.869
OMR2	0.877	OFR1	0.875
OMR3	0.901	OFR2	0.884
OMR4	0.882	OFR3	0.894
OMR5	0.881	OFR4	0.871
ERP1	0.895	ITA1	0.829
ERP2	0.866	ITA2	0.772
ERP3	0.881	ITA3	0.760

**Note.** This table shows the results of the factor analysis. All variables are defined in the S4 Appendix.

The factor loadings of the constructs indicated that all observed variables loaded strongly onto their respective factors. For instance, within the technological dimension, the loadings for Relative Advantage ranged between 0.843 and 0.891, Compatibility between 0.837 and 0.894, and Security between 0.852 and 0.882. Organizational constructs also showed high loadings, such as Higher Authority Support (0.829–0.912) and Monetary Resources (0.864–0.901), while environmental constructs like Rivalry Pressure (0.866–0.895) and Regulatory Support (0.796 0.894) demonstrated strong reliability as well. These findings validate the dimensional structure of the TOE framework, supporting its use in analyzing Blockchain adoption in the public sector. Accordingly, the results of the two models that were estimated to examine the influence of the TOE framework dimensions on the IABT using the SEM analysis are presented in Table 3. The first model evaluated the aggregate influence of the technological, organizational, and environmental perspectives, while the second model decomposed each perspective into its respective sub-factors.

**Table 3:** SEM results.

Panel A: Model One				
Parameter	Estimate	S.E.	C.R.	*p*-value
*IABT < --- Tech_Perspective*	0.398	0.073	5.468	***
*IABT < --- Org_Perspective*	0.308	0.068	4.498	***
*IABT < --- Env_Perspective*	0.346	0.068	5.057	***
**Panel B: Model Two**				
**Parameter**	**Estimate**	**S.E.**	**C.R.**	**p –value**
**Technological perspective**				
*IABT < --- Trust*	0.093	.030	3.109	***
*IABT < --- Compatibility*	0.277	.044	6.221	***
*IABT < --- Security*	0.099	.035	2.836	***
*IABT < --- Relative_Advantage*	0.012	.025	.493	0.622
**Organizational perspective**				
*IABT < --- High_Authority_Support*	0.093	.030	3.109	***
*IABT < --- Monetary_Resources*	0.114	.032	3.512	***
*IABT < --- Firm_Size*	0.046	.026	1.784	0.046
*IABT < --- IT_Resources*	0.979	1.205	.812	0.979
**Environmental perspective**				
*IABT < --- Rivalry_Pressure*	0.192	.033	5.900	***
*IABT < --- Regulatory_Support*	0.132	.028	4.642	***
*IABT < --- Business_Pressure*	0.041	.027	1.524	0.041

**Note.** This table shows the results of the influence of the technological, organizational and environmental factors on intention to adopt Blockchain in governmental operations in Sri Lanka. All variables are defined in the S4 Appendix. ***, **, and * indicate 1%, 5%, and 10% level of significance, respectively.

In Model One, all three TOE perspectives demonstrated significant and positive relationships with IABT. The technological perspective recorded a standardized coefficient of 0.398 (p < 0.001), the organizational perspective 0.308 (p < 0.001), and the environmental perspective 0.346 (p < 0.001). These results confirm that all three dimensions of the TOE framework are significant predictors of Blockchain adoption in the Sri Lankan governmental operations. This finding aligns with prior research emphasizing that technological readiness, organizational support, and environmental influences jointly drive technology adoption [61–65]. Therefore, the study supports Hypotheses 1, 2, and 3, confirming the applicability of the TOE model within the public sector operations the Sri Lankan context.

Model Two provides a more detailed examination by analyzing each sub-factor under the three perspectives. Within the technological perspective, Trust (β = 0.093, p < 0.01), Compatibility (β = 0.277, p < 0.001), and Security (β = 0.099, p < 0.01) were found to have positive and statistically significant effects on Blockchain adoption. However, Relative Advantage (β = 0.012, p = 0.622) was insignificant, indicating that while Blockchain offers efficiency and transparency, its perceived advantage over existing systems may not be fully recognized by public sector entities. In the organizational perspective, Higher Authority Support (β = 0.093, p < 0.01) and Monetary Resources (β = 0.114, p < 0.01) showed strong and significant positive relationships with IABT. This implies that the endorsement and involvement of senior management, along with sufficient financial resources, play crucial roles in facilitating Blockchain adoption in governmental institutions. Conversely, Firm Size (β = 0.046, p = 0.07) and IT Resources (β = 0.979, p > 0.05) demonstrated weak or insignificant effects. The latter finding suggests that while technical infrastructure is necessary, its presence alone is not sufficient for adoption without strategic leadership and financial support. For the environmental perspective, Rivalry Pressure (β = 0.192, p < 0.001) and Regulatory Support (β = 0.132, p < 0.001) exhibited strong positive relationships with IABT, indicating that external competition among agencies and a conducive regulatory environment significantly encourage adoption. However, Business Partner Pressure (β = 0.041, p > 0.05) was statistically insignificant, suggesting that external collaboration or partner influence currently has minimal effect on Blockchain adoption decisions within Sri Lankan government entities.

The results were further validated through multiple model fit indices, presented in Table 4. The chi-square values of 3452.558 (Model One) and 3502.431(Model Two) indicated model sensitivity to sample size; the CMIN/DF values of 3.206 (Model One) and 6.008 (Model Two) confirmed acceptable levels of model fit. Interestingly, the RMSEA values for both models were 0.000, indicating a close approximation between the theoretical and empirical models as the values below 0.05 [66].

**Table 4:** Goodness-of-fit results.

Goodness-of-fit indices	Model One	Model Two
Chi-square	3452.558	3502.431
**Absolute goodness of fit measure**		
CMIN/DF	3.206	6.008
RMSEA	0.000	0.000
**Incremental fit measure**		
CFI	0.677	0.436
IFI	0.680	0.441
TLI	0.662	0.390
**Parsimony fit measure**		
PCFI	0.647	0.403
PNFI	0.566	0.367

**Note.** This table shows the results of the goodness-of-fit.

Further, the Comparative Fit Index (CFI), Incremental Fit Index (IFI), and Tucker-Lewis Index (TLI) for Model One were 0.677, 0.680, and 0.662, respectively, reflecting moderate fit levels typical in behavioral research involving complex constructs. However, Model Two reported slightly lower fit indices (CFI = 0.436, IFI = 0.441, TLI = 0.390), which is expected given its greater complexity and inclusion of more granular variables. Although the incremental fit indices were lower, Model Two provided deeper insights into specific drivers of Blockchain adoption. The Parsimony Fit Indices (PCFI = 0.647, PNFI = 0.566) for Model One demonstrated a strong balance between model fit and simplicity, whereas Model Two (PCFI = 0.403, PNFI = 0.367) revealed the trade-off between model parsimony and analytical depth. Alternatively, an optimized model was developed by removing the insignificant variables to address the under fitting issue of Model Two. S5 Appendix shows the goodness of fit results of the optimized model, improving the credibility of SEM analysis, while supporting the interpretations of Model Two.

Overall, the SEM results indicate that all three TOE dimensions are significantly associated with intention to adopt Blockchain technology in Sri Lanka’s public sector. At the sub-factor level, trust, compatibility, security, higher authority support, monetary resources, rivalry pressure, and regulatory support showed significant positive relationships with adoption intention. By contrast, relative advantage, IT resources, and business partner pressure were not statistically significant, while firm size showed only weak support. These findings should therefore be interpreted as evidence regarding adoption intention and perceived organizational readiness rather than actual implementation outcomes.

The findings further indicate that Blockchain adoption in the Sri Lankan governmental operations is best understood as a multidimensional organizational process shaped by the joint influence of technological, organizational, and environmental conditions. From a TOE perspective, this means that adoption intention is not formed through technological characteristics alone, but through the interaction of institutional fit, organizational readiness, and environmental legitimacy [49–52]. The significance of all three TOE dimensions therefore suggests that Blockchain adoption in government is an institutionally embedded process in which administrative support structures and regulatory conditions matter alongside the properties of the technology itself.

The technological findings provide several important theoretical insights. Compatibility emerged as a significant predictor, suggesting that public sector institutions evaluate Blockchain primarily in terms of its fit with existing administrative systems and legacy infrastructure rather than as a stand-alone innovation. This is theoretically important because government organizations typically operate within rigid procedural environments where interoperability and continuity of service are essential [32]. Moreover, the significance of trust and security indicates that technological adoption in government is strongly tied to governance reliability, accountability, and risk reduction. Since public agencies handle sensitive citizen and state information, the perceived trustworthiness and security of Blockchain become central to adoption intention [33,18,40,41]. In contrast, the non-significance of relative advantage suggests that perceived efficiency gains alone are insufficient to motivate adoption in this context, implying that adoption decisions are driven less by technological appeal and more by institutional fit and uncertainty reduction.

The organizational findings further refine the TOE framework for public administration. Higher authority support and monetary resources were both significant, indicating that Blockchain adoption in government depends heavily on hierarchical authorization and formal resource commitment. This is consistent with prior studies showing that leadership commitment mobilizes resources, reduces organizational resistance, and creates the legitimacy necessary for technological change [35]. Similarly, adequate financial and technical resources influence the feasibility and sustainability of adoption [36]. These findings suggest that organizational readiness in the public sector should not be understood simply as technical preparedness, but as the presence of administrative sponsorship and budgetary capacity. By contrast, the weak or insignificant role of IT resources indicates that infrastructure alone does not ensure adoption intention unless it is accompanied by strategic leadership and institutional prioritization.

The environmental findings are also theoretically important because they show that public-sector Blockchain adoption is shaped not only by internal readiness, but also by external legitimacy conditions. Rivalry pressure and regulatory support both had significant positive effects, indicating that adoption intention increases when agencies perceive modernization pressure from peer institutions and when the broader regulatory environment provides clarity and support [37,53]. This is especially important in public administration because technologies such as Blockchain require not only technical feasibility but also legal and policy legitimacy, particularly where privacy, accountability, and data governance are concerned [40,41]. Our results also support the recent findings of [67] showing that when adopting Blockchain-enabled innovations in public-sector operations in developing economies, the establishment of formal regulatory legitimacy acts as an essential institutional anchor that mitigates perceived administrative risk and directly cultivates institutional trust across fragmented government networks. The insignificance of business partner pressure suggests that Blockchain adoption in the Sri Lankan governmental operations is not yet being driven by external partner demand or collaborative ecosystem pressure, but rather by internal administrative priorities and state-centered modernization dynamics. These findings are especially meaningful in the context of Sri Lanka as a developing-country public sector, where public institutions face infrastructure gaps, uneven digital readiness, limited trained personnel, and insufficient regulatory clarity [19,20,42]. More broadly, research on Blockchain adoption in government remains limited, especially in developing-country settings [4,6,26].

Taken together, these findings suggest that the TOE dimensions do not operate with equal weight in this context. Instead, Blockchain adoption intention in the Sri Lankan governmental operations appears to be structured more strongly by compatibility, trust, security, higher authority support, monetary resources, rivalry pressure, and regulatory support than by relative advantage, IT resources, or business partner pressure. Theoretically, this refines the TOE framework by showing that in a developing-country public-sector context, institutional alignment and governance readiness are more decisive than technological attractiveness alone [49–52].

## 5. Conclusion

This study examined how technological, organizational, and environmental factors are associated with intention to adopt Blockchain technology in the Sri Lankan governmental operations. Using the TOE framework, the findings provide empirical support for all three proposed hypotheses. Specifically, technological factors showed a significant positive relationship with adoption intention, thereby supporting Hypothesis 1; organizational factors also showed a significant positive relationship, thereby supporting Hypothesis 2; and environmental factors significantly influenced adoption intention, thereby supporting Hypothesis 3. These findings indicate that Blockchain adoption in the Sri Lankan public sector is shaped by the combined influence of technological readiness, organizational support, and environmental conditions rather than by any single factor in isolation.

At a more specific level, trust, compatibility, security, higher authority support, monetary resources, rivalry pressure, and regulatory support emerged as significant predictors of adoption intention. In contrast, relative advantage, IT resources, and business partner pressure were not statistically significant, while firm size showed only limited support. These results suggest that Blockchain adoption intention in the Sri Lankan governmental operations depends more strongly on institutional fit, leadership support, financial readiness, and regulatory legitimacy than on perceived technological advantage alone. These findings should be interpreted as evidence regarding adoption intention and perceived organizational readiness, rather than as direct evidence of actual implementation outcomes or realized governance improvements. Although Blockchain may potentially contribute to transparency, efficiency, and trust in public administration, those downstream effects were not directly measured in this study. Therefore, the contribution of this research is to clarify the factors associated with Blockchain adoption intention in the Sri Lankan public sector, not to demonstrate the actual impact of Blockchain implementation on administrative performance or governance outcomes.

The implications of this study are substantial for policymakers, senior government officials, and IT managers. On the one hand, policymakers, the findings stress the need to establish clear regulatory frameworks that align Blockchain’s decentralized features with the national data protection laws such as, Sri Lanka’s Personal Data Protection Act (2022), and international standards such as ISO/IEC 27001, the GDPR. On the other hand, policymakers should also address scalability and performance concerns through guidelines that facilitate inter-operability between Blockchain systems and existing public sector platforms. For government officials, the study highlights that leadership engagement and inter-departmental collaboration are vital to overcome bureaucratic inertia and foster a culture of innovation. By promoting cross-agency partnerships and knowledge sharing, public sector leaders can create an integrated environment conducive to large-scale Blockchain deployment.

From a managerial perspective, this study underscores the necessity of sufficient financial investment and resource planning. In particular, budgetary provisions should be allocated specifically for Blockchain initiatives, covering infrastructure, training, and system maintenance. Moreover, the partnerships with international development organizations such as the World Bank or UNDP can further support funding for pilot programs that test Blockchain’s applicability in various administrative functions. For IT managers, technical readiness remains essential while staff training, risk-management protocols, and pilot implementations in limited departments will help identify operational challenges before scaling. These steps ensure smooth technology adoption while minimizing disruptions to critical services.

Based on the findings of this study, several recommendations are proposed for effective Blockchain adoption in the Sri Lankan government institutions. First, fostering inter-departmental collaboration is critical to build a cohesive and standardized approach to Blockchain implementation. Second, the development of tailored risk-management frameworks is necessary to address Blockchain’s unique security and privacy concerns. Accordingly, a comprehensive Blockchain Adoption Framework is provided in the S6 Appendix, offering a strategic guidance based on the TOE dimensions. Complementing this, a practical Blockchain Implementation Checklist is also presented in the S7 Appendix, outlining step-by-step procedures for effective Blockchain adoption across government functions. Third, conducting pilot projects in smaller departments will allow public sector organizations to evaluate Blockchain’s practical implications, refine procedures, and build capacity before full-scale deployment. Finally, strengthening the regulatory environment is essential to ensure that Blockchain initiatives align with existing laws and promote transparency, accountability, and efficiency.

While this study provides valuable insights, certain limitations must be recognized. The research was confined to Sri Lanka’s public sector, which may restrict the generalizability of findings to other contexts. A further limitation is that the study relied on a single key-informant group, namely the Grade I ICT officers in the Sri Lankan public sector institutions. Although these respondents are well positioned to evaluate institutional technology readiness and implementation conditions, Blockchain adoption in practice may also be shaped by other actors, including department heads, legal officers, policymakers, ordinary civil servants, and external stakeholders. Future research should therefore adopt a multi-respondent design that includes multiple organizational levels and stakeholder groups to provide a more comprehensive assessment of the public sector Blockchain adoption readiness. In addition, the dependent variable in this study was intention to adopt Blockchain technology rather than actual implementation or post-adoption performance. Therefore, the findings should not be interpreted as direct evidence of realized governance outcomes such as reduced corruption, faster administrative processing, or increased citizen trust. Additionally, this study focused solely on Blockchain and did not examine other emerging technologies such as smart contracts or decentralized applications that could complement its adoption. In particular, as Blockchain technology continues to evolve, future research should expand its scope by evaluating the empirical and technical convergence of emerging artificial intelligence paradigms with decentralized architectures. Rather than treating machine learning as a generalized concept, future studies should specifically investigate the operational value of integrating Large Language Models (LLMs) within Blockchain-based government data governance networks. [68] demonstrate thar in multi-actor transaction ecosystems, a collaborative architecture of LLM and Blockchain offers significant cross-domain migration value for public administration in developing economies. Hnece, future research should model how LLM text processing capabilities can work synergistically with distributed ledgers to automate complex administrative compliance inspections, dynamically generate multilingual clauses for governmental smart contracts, and execute cross-departmental semantic data alignment during multi-agency information exchanges. Testing these integrated data governance paths represents a vital next step for upgrading public sector digital transformation research from broad descriptive concepts to precise structural roadmaps.

## Supporting information

S1 Appendix Public sector institutions in the sampling frame. (DOCX)

S2 Appendix Questionnaire. (DOCX)

S3 Data Data used for the analysis of this study. (XLSX)

S4 Appendix Latent variables. (DOCX)

S5 Appendix Goodness-of-fit results of the optimized model. (DOCX)

S6 Appendix Blockchain adoption framework. (DOCX)

S7 Appendix Blockchain adoption checklist. (DOCX)
