# Supplementary material for: Enhancing blockchain technology adoption in governmental operations: A comprehensive framework for user adoption
Source: PLoS One. 2026 Jul 6;21(7):e0352781. doi: 10.1371/journal.pone.0352781 (PMC13336220; doi:10.1371/journal.pone.0352781)
Supplement: S1 Appendix — (DOCX) [file pone.0352781.s001.docx]

| **S1 Appendix. Public sector institutions in the sampling frame.** | |
| --- | --- |
| **Institutional category** | **Institutions represented in the sampling frame** |
| Central executive offices | President’s Office |
|  | Prime Minister’s Office |
|  | Office of the Cabinet of Ministers |
| Major service ministries and service-related units | Ministry of Education |
|  | Ministry of Health – Medical Supply Unit |
|  | Epidemiology Unit; Family Health Bureau |
| Revenue, finance, and administrative institutions | Ministry of Finance - Department of |
|  | Information Technology Management |
|  | Department of State Account |
|  | Department of Inland Revenue |
|  | Department of Import and Export Control of Sri Lanka |
| Operational and citizen-service departments | Ministry of Foreign Affairs – Consular Affairs Division |
|  | Department of Examinations |
|  | Department of Railways |
|  | Department of Elections |
|  | Department of Post |
|  | Department of Examination |
|  | Ministry of Fisheries |
|  | Department of Pensions |
|  | Department of Registration of Persons |
| Data, statistics, and technical agencies | Department of Census and Statistics |
|  | Ministry of Digital Economy |
| Other ministries and specialized  Departments | Ministry of Public Administration and Local Government |
|  | Department of Surveys |
|  | Department of Motor Traffic |
|  | Department of Irrigations |
|  | Department of Immigration and Emigration |
|  |  |
| **Note.** Compiled from the Minute of the Sri Lanka Information and Communication Technology Service (Gazette Extraordinary No. 1894/26, 26 December 2014). | |
