# Supplementary material for: Enhancing blockchain technology adoption in governmental operations: A comprehensive framework for user adoption
Source: PLoS One. 2026 Jul 6;21(7):e0352781. doi: 10.1371/journal.pone.0352781 (PMC13336220; doi:10.1371/journal.pone.0352781)
Supplement: S2 Appendix — (DOCX) [file pone.0352781.s002.docx]

**S2 Appendix. Questionnaire.**

**Enhancing Blockchain Technology Adoption in Governmental Operations:**

**A Comprehensive Framework for User Adoption**

Dear Respondent,

The purpose of this survey is to gather insights from the Directors in the Grade I - IT service in the Sri Lankan public sector organizations on the factors influencing the adoption of Blockchain technology in governmental operations in Sri Lanka.

The survey will take approximately 10-20 minutes to complete. Please be assured that this is purely for academic purposes, and all responses will be stored securely. No personal data will be collected, and you have the right to withdraw from the survey at any time. Your responses will remain confidential and will be used solely for academic research.

Thank you for your participation.

| **Demographic factors** | | | | | | | | | | |
| --- | --- | --- | --- | --- | --- | --- | --- | --- | --- | --- |
| **1. Age** | |  |  |  | | | |  | | |
|  | 20-30 years |  |  |  | | | |  | | |
|  | 30-40 years |  |  |  | | | |  | | |
|  | 40-50 years |  |  |  | | | |  | | |
|  | Above 50 years | |  |  | | | |  | | |
| **2. Gender** |  |  |  |  | | | |  | | |
|  | Male |  |  |  | | | |  | | |
|  | Female |  |  |  | | | |  | | |
|  |  |  |  |  | | | |  | | |
| **3. Educational Qualification** | | | | | | | |  | | |
|  | Bachelor’s Degree | |  |  | | | |  | | |
|  | Master’s Degree | |  |  | | | |  | | |
|  | MPhil |  |  |  | | | |  | | |
|  |  |  |  |  | | | |  | | |
| **4. Years of Service** | |  |  |  | | | |  | | |
|  | Less than 5 years | |  |  | | | |  | | |
|  | 5-10 years |  |  |  | | | |  | | |
|  | More than 10 years | |  |  | | | |  | | |
|  |  |  |  |  | | | |  | | |
| **Please select only ONE response to each question from Question 05 to Question 16.** | | | | | | | | | | |
|  | **1= Strongly Disagree** | |  |  | | | |  | | |
|  | **2 = Disagree** | |  |  | | | |  | | |
|  | **3 = Neutral** |  |  |  | | | |  | | |
|  | **4 = Agree** |  |  |  | | | |  | | |
|  | **5 = Strongly Agree** | |  |  | | | |  | | |
|  |  |  |  |  | | | |  | | |
| **5.** **Relative Advantage** | | | | | |  |  | |  |  |
|  | | | | | **1** | **2** | **3** | | **4** | **5** |
| Blockchain technology will improve the efficiency of government processes. | | | | |  |  |  | |  |  |
| Blockchain adoption will reduce operational costs in our department. | | | | |  |  |  | |  |  |
| Blockchain will enhance the productivity of government functions. | | | | |  |  |  | |  |  |
| Blockchain will introduce greater flexibility to governmental operations. | | | | |  |  |  | |  |  |
|  | | | | |  |  |  | |  |  |
| **6. Trust** | | | | |  |  |  | |  |  |
| I am confident that Blockchain technology will securely store government data. | | | | |  |  |  | |  |  |
| Third-party access to government data is a concern when using Blockchain technology. | | | | |  |  |  | |  |  |
| There is a risk of unauthorized access to government data stored in the cloud through Blockchain. | | | | |  |  |  | |  |  |
|  | | | | |  |  |  | |  |  |
| **7. Compatibility** | | | | |  |  |  | |  |  |
| Blockchain technology aligns with the existing workflow in my department. | | | | |  |  |  | |  |  |
| I would implement blockchain if it meets the specific needs of my department. | | | | |  |  |  | |  |  |
| Blockchain fits well with our current governmental operations. | | | | |  |  |  | |  |  |
| Blockchain technology may not be compatible with every aspect of our operations. | | | | |  |  |  | |  |  |
|  | | | | |  |  |  | |  |  |
| **8. Security** | | | | |  |  |  | |  |  |
| Traditional IT systems offer better security than Blockchain. | | | | |  |  |  | |  |  |
| Security concerns significantly influence our decision to adopt Blockchain. | | | | |  |  |  | |  |  |
| The potential risks of using Blockchain outweigh its benefits. | | | | |  |  |  | |  |  |
|  | | | | |  |  |  | |  |  |
| **9. Higher Authority Support** | | | | |  |  |  | |  |  |
| Support from senior management is crucial for Blockchain technology adoption in our department. | | | | |  |  |  | |  |  |
| Decisions to adopt Blockchain technology are heavily influenced by higher-level authorities. | | | | |  |  |  | |  |  |
| Senior management must be involved in decision-making regarding Blockchain adoption. | | | | |  |  |  | |  |  |
| The role of senior officials is vital in reviewing recommendations for Blockchain adoption. | | | | |  |  |  | |  |  |
| As a decision-maker, I would prefer Blockchain technology for our department. | | | | |  |  |  | |  |  |
| **10. Firm Size** | | | | |  |  |  | |  |  |
| Larger ministries and departments will benefit more from Blockchain due to their ability to invest. | | | | |  |  |  | |  |  |
| Smaller departments could adopt Blockchain through a pay-per-use model. | | | | |  |  |  | |  |  |
| Smaller departments may avoid Blockchain technology due to resource constraints. | | | | |  |  |  | |  |  |
|  | | | | |  |  |  | |  |  |
| **11. Monetary Resources** | | | | |  |  |  | |  |  |
| Financial resources are a significant factor in adopting Blockchain technology in government. | | | | |  |  |  | |  |  |
| Only financially strong government institutions can afford Blockchain technology. | | | | |  |  |  | |  |  |
| Larger government bodies are better positioned to adopt Blockchain due to their financial stability. | | | | |  |  |  | |  |  |
| Financially secure departments should prioritize Blockchain adoption. | | | | |  |  |  | |  |  |
| Smaller departments may not gain as much from Blockchain technology due to limited budgets. | | | | |  |  |  | |  |  |
|  | | | | |  |  |  | |  |  |
| **12. IT Resources** | | | | |  |  |  | |  |  |
| Our department needs strong technological infrastructure and skilled staff to support Blockchain adoption. | | | | |  |  |  | |  |  |
| The human resources within the department need sufficient knowledge to adopt Blockchain technology. | | | | |  |  |  | |  |  |
| Reliable internet connectivity is essential for the effective utilization of Blockchain. | | | | |  |  |  | |  |  |
| Strong IT resources will improve the efficiency of our governmental systems. | | | | |  |  |  | |  |  |
|  | | | | |  |  |  | |  |  |
| **13. Rivalry Pressure** | | | | |  |  |  | |  |  |
| Blockchain adoption is essential to enhance data accuracy in government services. | | | | |  |  |  | |  |  |
| Blockchain will improve the efficiency of government operations. | | | | |  |  |  | |  |  |
| Implementing Blockchain will provide our department with a competitive edge. | | | | |  |  |  | |  |  |
| Blockchain helps in maintaining control over the data sharing process. | | | | |  |  |  | |  |  |
|  | | | | |  |  |  | |  |  |
| **14. Business Partner Pressure** | | | | |  |  |  | |  |  |
| I would adopt Blockchain technology to improve collaboration with external partners. | | | | |  |  |  | |  |  |
| Our department’s partners are recommending  Blockchain adoption. | | | | |  |  |  | |  |  |
| Blockchain adoption will strengthen trust between our department and external partners. | | | | |  |  |  | |  |  |
| External partners of our department oppose Blockchain adoption. | | | | |  |  |  | |  |  |
| Our department’s partners support the decisions made regarding Blockchain. | | | | |  |  |  | |  |  |
|  | | | | |  |  |  | |  |  |
| **15. Regulatory Pressure** | | | | |  |  |  | |  |  |
| I would adopt Blockchain technology if it complies with national regulations. | | | | |  |  |  | |  |  |
| I would implement Blockchain technology if it aligns with our technical guidelines. | | | | |  |  |  | |  |  |
| Existing regulations are insufficient to safeguard data stored in Blockchain systems. | | | | |  |  |  | |  |  |
| It is essential that Blockchain systems comply with national laws on data protection. | | | | |  |  |  | |  |  |
|  | | | | |  |  |  | |  |  |
| **16. Intention to Adopt Blockchain Technology** | | | | |  |  |  | |  |  |
| If Blockchain technology is accessible, I intend to adopt it in my department. | | | | |  |  |  | |  |  |
| I am willing to implement Blockchain technology if it is available. | | | | |  |  |  | |  |  |
| I plan to use Blockchain technology in future government operations. | | | | |  |  |  | |  |  |
