# Supplementary material for: Enhancing blockchain technology adoption in governmental operations: A comprehensive framework for user adoption
Source: PLoS One. 2026 Jul 6;21(7):e0352781. doi: 10.1371/journal.pone.0352781 (PMC13336220; doi:10.1371/journal.pone.0352781)
Supplement: S4 Appendix — (DOCX) [file pone.0352781.s004.docx]

| **S4 Appendix. Latent variables.** | | | |
| --- | --- | --- | --- |
| **Latent variable** | **Item** | | **Source** |
| Relative Advantage | Blockchain technology will improve the efficiency of government processes. | TRA1 | [1] |
|  | Blockchain adoption will reduce operational costs in our department. | TRA2 |  |
|  | Blockchain will enhance the productivity of government functions. | TRA3 |  |
|  | Blockchain will introduce greater flexibility to governmental operations. | TRA4 |  |
| Trust | I am confident that Blockchain technology  will securely store government data. | TTR1 | [2], [3] |
|  | Third-party access to government data is a concern when using Blockchain technology. | TTR2 |  |
|  | There is a risk of unauthorized access to government data stored in the cloud through Blockchain. | TTR3 |  |
| Security | Traditional IT systems offer better security than Blockchain. | TSE1 | [2], [3] |
|  | Security concerns significantly influence  our decision to adopt Blockchain. | TSE2 |  |
|  | The potential risks of using Blockchain  outweigh its benefits. | TSE3 |  |
| Higher Authority Support | Support from senior management is crucial for Blockchain technology adoption in our  department. | OHAC1 | [2], [3], [4] |
|  | Decisions to adopt Blockchain technology are heavily influenced by higher-level authorities. | OHAC2 |  |
|  | Senior management must be involved in decision-making regarding Blockchain adoption. | OHAC3 |  |
|  | The role of senior officials is vital in reviewing recommendations for Blockchain adoption. | OHAC4 |  |
|  | As a decision-maker, I would prefer Blockchain technology for our department. | OHAC5 |  |
| Firm Size | Larger ministries and departments will benefit more from Blockchain due to their ability to invest. | OOFS1 | [5]  *Continued…* |
|  | Smaller departments could adopt Blockchain through a pay-per-use model. | OOFS2 |  |
|  | Smaller departments may avoid Blockchain technology due to resource constraints. | OOFS3 |  |
| Monetary Resources | Financial resources are a significant factor  in adopting Blockchain technology in government. | OMR1 | [6] |
|  | Only financially strong government institutions can afford Blockchain technology. | OMR2 |  |
|  | Larger government bodies are better positioned to adopt Blockchain due to their financial stability. | OMR3 |  |
|  | Financially secure departments should prioritize Blockchain adoption. | OMR4 |  |
|  | Smaller departments may not gain as much from Blockchain technology due to limited budgets. | OMR5 |  |
| Rivalry Pressure | Blockchain adoption is essential to enhance  data accuracy in government services. | ERP1 | [7] |
|  | Blockchain will improve the efficiency of government operations. | ERP2 |  |
|  | Implementing Blockchain will provide our department with a competitive edge. | ERP3 |  |
|  | Blockchain helps in maintaining control  over the data-sharing process. | ERP4 |  |
| Business Partner Pressure | I would adopt Blockchain technology to improve collaboration with external partners. | EBP1 | [8], [9] |
|  | Our department’s partners are recommending Blockchain adoption. | EBP2 |  |
|  | Blockchain adoption will strengthen trust  Between our department and external partners. | EBP3 |  |
|  | External partners of our department oppose Blockchain adoption. | EBP4 |  |
|  | Our department’s partners support the  decisions made regarding Blockchain. | EBP5 |  |
| Regulatory Support | I would adopt Blockchain technology if it  complies with national regulations. | ERS1 | [10]  *Continued…* |
|  | I would implement Blockchain technology  if it aligns with our technical guidelines. | ERS2 |  |
|  | Existing regulations are insufficient to safeguard data stored in Blockchain systems. | ERS3 |  |
|  | It is essential that Blockchain systems comply with national laws on data protection. | ERS4 |  |
| IT Resources | Our department needs strong technological  infrastructure and skilled staff to support Blockchain adoption. | OOFS1 | [11] |
|  | The human resources within the department  Need sufficient knowledge to adopt Blockchain technology. | OOFS2 |  |
|  | Reliable internet connectivity is essential for the effective utilization of Blockchain. | OOFS3 |  |
|  | Strong IT resources will improve the  efficiency of our governmental systems. |  |  |
| Intention to Adopt Blockchain Technology | If Blockchain technology is accessible, I  intend to adopt it in my department. | ITA1 | [12] |
|  | I am willing to implement Blockchain  technology if it is available. | ITA2 |  |
|  | I plan to use Blockchain technology in  future government operations. | ITA3 |  |
|  |  |  |  |
| **Note.** Adopted from [54]. | | | |

**References**

[1] G. C. Moore and I. Benbasat, “Development of an instrument to measure the perceptions of adopting an information technology innovation,” *Information Systems Research*, vol. 2, no. 3, pp. 192–222, 1991. doi: 10.1287/isre.2.3.19

[2] P. R. Kumar, P. H. Raj, and P. Jelciana, “Exploring data security issues and solutions in cloud computing,” *Procedia Computer Science*, vol. 125, pp. 691–697, 2018.

[3] K. Zhu, K. Kraemer, and S. Xu, "The process of innovation assimilation by firms in different countries: A technology diffusion perspective on e-business," Management Science, vol. 52, no. 10, pp. 1557-1576, Oct 2006.

[4] G. Premkumar and M. Roberts, “Adoption of new information technologies in rural small businesses,” *Omega*, vol. 27, no. 4, pp. 467–484, 1999. doi: 10.1016/S0305-0483(98)00071-1

[5] R. Vandaie, “The role of organizational knowledge management in successful ERP implementation projects,” *Knowledge-Based Systems*, vol. 21, no. 8, pp. 920–926, 2008. doi: 10.1016/j.knosys.2008.04.001

[6] V. Chittipaka, S. Kumar, U. Sivarajah, J. L. H. Bowden, and M. M. Baral, “Blockchain technology for supply chain operating in emerging markets: An empirical examination of the technology-organization-environment (TOE) framework,” *Annals of Operations Research*, vol. 327, pp. 465–492, 2023. doi: 10.1007/s10479-022-04801-5

[7] M. S. Satar and G. Alarifi, “Factors of E-Business Adoption in Small and Medium Enterprises: Evidence from Saudi Arabia,” *Human Behavior and Emerging Technologies*, vol. 2022, pp. 1–13, 2022. doi: 10.1155/2022/2445624

[8] T. I. Akaba, A. Norta, C. Udokwu, and D. Draheim, “A framework for the adoption of blockchain-based e-procurement systems in the public sector: A case study of Nigeria,” in *Proc. 19th IFIP Conf. e-Business, e-Services and e-Society*, South Africa, 2020.

[9] H. Taherdoost, “Legal, regulatory, and ethical considerations in e-business,” in *E-Business Essentials* (EAI/Springer Innovations in Communication and Computing). Cham, Switzerland: Springer, 2023, pp. 379–402.

[10] B. Gupta, S. Dasgupta, and A. Gupta, “Adoption of ICT in a government organization in a developing country: An empirical study,” *The Journal of Strategic Information Systems*, vol. 17, no. 2, pp. 140–154, 2008. doi: 10.1016/j.jsis.2007.12.004

[11] D. Cagigas, J. Clifton, D. D. Fuentes, M. Fernández-Gutiérrez, and C. Harpes, “Blockchain in government: Toward an evaluation framework,” *Policy Design and Practice*, vol. 6, no. 4, pp. 397–414, 2023. doi: 10.1080/25741292.2023.2230702

[12] J. C. Nunnally, *Psychometric Theory*, 3rd ed. New York, NY, USA: McGraw-Hill, 1994.
