# Supplementary material for: Enhancing blockchain technology adoption in governmental operations: A comprehensive framework for user adoption
Source: PLoS One. 2026 Jul 6;21(7):e0352781. doi: 10.1371/journal.pone.0352781 (PMC13336220; doi:10.1371/journal.pone.0352781)
Supplement: S5 Appendix — (DOCX) [file pone.0352781.s005.docx]

| **S5 Appendix. Goodness-of-fit results of the optimized model.** | |
| --- | --- |
| **Goodness-of-Fit Indices** | **Optimized Model** |
| **Overall Fit** |  |
| Chi-square | 2939.620 |
| **Absolute Goodness-of-Fit Measures** |  |
| CMIN/DF | 2.854 |
| RMSEA | 0.000 |
| **Incremental Fit Measures** |  |
| CFI | 0.723 |
| IFI | 0.736 |
| TLI | 0.708 |
| **Parsimony Fit Measures** |  |
| PCFI | 0.682 |
| PNFI | 0.594 |
|  |  |
| **Note.** This table shows the goodness-of-fit results of the optimized model. | |
