# Supplementary material for: Enhancing blockchain technology adoption in governmental operations: A comprehensive framework for user adoption
Source: PLoS One. 2026 Jul 6;21(7):e0352781. doi: 10.1371/journal.pone.0352781 (PMC13336220; doi:10.1371/journal.pone.0352781)
Supplement: S6 Appendix — (DOCX) [file pone.0352781.s006.docx]

| **S6 Appendix. Blockchain adoption framework.** | | |
| --- | --- | --- |
| **Part One – Key factors to be addressed** | | |
| **Factor** | **Action** | **Goal** |
| Trust | Build trust in Blockchain technology by implementing pilot projects in non-sensitive but impactful government areas (e.g., public financial management, citizen services). | Create confidence in the technology's reliability and security features through small, low-risk projects. |
| Compatibility | Ensure Blockchain solutions integrate with existing government IT infrastructure (e.g., Lanka Government Cloud, e-government platforms like Lanka Gate).  Implement middleware solutions where necessary to ensure compatibility. | Seamlessly incorporate Blockchain without disrupting existing workflows. |
| Security | Conduct comprehensive security assessments to ensure that Blockchain implementations meet Sri Lanka's national data security standards (e.g., CERT\|CC).  Introduce encryption, identity management, and secure access protocols. | Safeguard sensitive governmental data from unauthorized access or breaches, leveraging Blockchain’s cryptographic strengths. |
| High Authority Support | Secure support from high-level government officials (e.g., from the Ministry of Digital Infrastructure and ICTA) to champion Blockchain initiatives. | Ensure Blockchain projects have leadership backing and are prioritized in national digital transformation strategies. |
| Monetary Resources | Allocate dedicated budgets for Blockchain projects, covering technology procurement, development, and maintenance.  Explore partnerships with international organizations (e.g., UNDP, World Bank) to secure funding for pilot programs. | Ensure sustainable funding for Blockchain adoption and reduce the financial burden on smaller governmental bodies. |
| Firm Size | Tailor Blockchain solutions to the needs of different-sized government departments.  Large ministries (e.g., Ministry of Finance) might deploy complex Blockchain applications, while smaller departments can adopt simpler, more cost-effective solutions. | Scale Blockchain adoption to fit the resource availability and needs of each governmental unit.  *Continued…* |
| Rivalry Pressure | Foster competition between government departments by recognizing early adopters and successful Blockchain projects. | Create a sense of urgency and encourage departments to innovate and adopt Blockchain as part of their modernization efforts. |
| Regulatory Pressure | Ensure Blockchain implementations comply with Sri Lanka’s legal and regulatory frameworks, such as the Personal Data Protection Act and cybersecurity laws.  Work with ICTA and other regulatory bodies to develop specific Blockchain guidelines. | Maintain compliance and address legal concerns regarding data ownership, privacy, and transparency. |
| **Part Two – Implementation guidelines** | | |
| **Activity** | **Action** | **Goal** |
| Phased Implementation | Start with small-scale pilot projects (e.g., e- procurement, health records), test their feasibility, and gather feedback.  Based on the outcomes, scale up to larger departments or interdepartmental projects. | Mitigate risk by piloting Blockchain in specific departments and expand gradually based on success. |
| Staff Training and Capacity Building | Organize nationwide training programs for ICT officers and decision-makers to familiarize them with Blockchain’s operational and strategic aspects. | Build internal capacity to manage and maintain Blockchain systems across all levels of government. |
| Collaboration and Partnerships | Establish partnerships with local universities (e.g., University of Moratuwa), international Blockchain experts, and private-sector companies to facilitate knowledge sharing and support. | Leverage external expertise and technology providers to accelerate Blockchain adoption. |
| Monitoring and Evaluation  o | Set clear performance metrics (e.g., cost reductions, efficiency gains, transparency improvements) to assess the success of Blockchain implementations.  Monitor projects regularly and adjust strategies based on real-time data. | Ensure continuous improvement and data-driven decision-making in Blockchain adoption. |
| Risk Management | Develop a comprehensive risk management plan that addresses potential challenges such as data security risks, system failures, or regulatory issues. | Minimize risks and have contingency plans ready for addressing unexpected  problems. |
