# Supplementary material for: Enhancing blockchain technology adoption in governmental operations: A comprehensive framework for user adoption
Source: PLoS One. 2026 Jul 6;21(7):e0352781. doi: 10.1371/journal.pone.0352781 (PMC13336220; doi:10.1371/journal.pone.0352781)
Supplement: S7 Appendix — (DOCX) [file pone.0352781.s007.docx]

| **S7 Appendix. Blockchain adoption checklist.** | |
| --- | --- |
| **Factor** | **Checklist Items** |
| Trust | - Implement pilot projects in sectors like healthcare or public finance to build trust in Blockchain’s transparency and immutability features. - Use blockchain’s security features (e.g., encryption) to address concerns about sensitive data handling in government operations. |
| Compatibility | - Ensure that Blockchain solutions are compatible with Sri Lanka’s existing e-government infrastructure (e.g., Lanka Government Cloud, Lanka Gate). - Ensure Blockchain platforms can integrate smoothly with legacy systems used by various government departments. |
| Security | - Conduct a comprehensive security assessment to align Blockchain implementations with Sri Lanka’s national cybersecurity policies (e.g., CERT\|CC guidelines). - Implement multi-layered security protocols (e.g., encryption, secure access) to safeguard classified government data. |
| High Authority Support | - Obtain support from senior government leadership (e.g., Ministry of Digital Infrastructure) to ensure political and organizational backing for Bblockchain initiatives. - Establish a government task force or committee to oversee the nationwide rollout of Blockchain in government operations. |
| Monetary Resources | - Secure sufficient budget allocations for Blockchain-related infrastructure upgrades, implementation, and training. - Seek collaboration with international development organizations (e.g., UNDP, World Bank) for technical and financial support in Blockchain adoption. |
| Firm Size | - Customize Blockchain adoption strategies based on the size of the government entity. Large ministries might have the capacity for complex implementations, while smaller departments could adopt simpler solutions. - Smaller government departments can explore cost-effective Blockchain models like pay-per-use services. |
| Rivalry Pressure | - Encourage competition among ministries and departments by showcasing successful Blockchain projects in local government agencies. - Offer incentives (e.g., national recognition, resource allocation) for early adopters of Blockchain solutions in the public sector. |
| Regulatory Pressure | - Ensure that Blockchain adoption complies with national regulatory frameworks, including data protection laws (e.g., Sri Lanka’s Personal Data Protection Act). - Collaborate with regulatory bodies (e.g., ICTA, Central Bank of Sri Lanka) to develop clear policies and guidelines that promote Blockchain adoption   while ensuring legal compliance. |

**Blockchain Adoption Checklist for Organizations**

Factor: Trust

1. Pilot Projects:

☐ Identify non-sensitive areas such as supply chain tracking or internal data management to implement pilot blockchain projects.

☐ Share case studies and success stories to build trust among stakeholders and employees.

2. Transparency:

☐ Utilize blockchain's immutability to create transparent audit trails for critical processes.

☐ Educate stakeholders on how blockchain enhances accountability and reduces fraud.

Factor: Compatibility

1. Infrastructure Assessment:

☐ Conduct a thorough audit of existing IT systems to ensure blockchain solutions align with current infrastructure.

☐ Plan for middleware development to bridge gaps between blockchain platforms and legacy systems.

2. Cloud Integration:

☐ Ensure compatibility with cloud-based solutions like AWS, Azure, or private clouds used by the organization.

Factor: Security

1. Data Protection:

☐ Implement advanced encryption protocols to secure sensitive data.

☐ Establish multi-factor authentication (MFA) for access to blockchain networks.

2. Threat Mitigation:

☐ Conduct regular security audits to identify vulnerabilities.

☐ Use blockchain’s consensus mechanisms to prevent unauthorized changes to records.

Factor: Leadership and Stakeholder Support

1. Executive Sponsorship:

☐ Secure buy-in from senior leadership to champion blockchain initiatives.

☐ Develop a clear business case outlining ROI and efficiency gains.

2. Stakeholder Engagement:

☐ Involve key stakeholders from various departments during the planning and implementation phases.

☐ Conduct regular updates and workshops to maintain engagement.

Factor: Budget and Funding

1. Resource Allocation:

☐ Allocate funds for research, development, and staff training.

☐ Explore cost-sharing partnerships with technology vendors or industry consortia.

2. External Funding:

☐ Apply for grants from industry bodies or international organizations supporting technology innovation.

Factor: Regulatory Compliance

1. Legal Framework:

☐ Consult with legal experts to ensure blockchain implementations comply with local and international laws.

☐ Monitor regulatory changes and adapt blockchain strategies accordingly.

2. Data Governance:

☐ Establish clear data ownership policies for blockchain transactions.

☐ Ensure compliance with data privacy regulations like GDPR or HIPAA.

Factor: Scalability

1. Flexible Design:

☐ Design blockchain solutions that can scale as the organization grows.

☐ Choose platforms supporting modular or hybrid blockchain approaches for flexibility.

2. Interoperability:

☐ Implement solutions that integrate seamlessly with other blockchain systems or external networks.

Factor: Employee Training and Change Management

1. Training Programs:

☐ Conduct workshops to educate employees on blockchain basics and applications.

☐ Provide specialized training for IT staff on blockchain deployment and maintenance.

2. Change Management:

☐ Develop a communication plan to address employee concerns about adopting blockchain.

☐ Highlight benefits such as reduced workloads and increased transparency.

Factor: Collaboration and Partnerships

1. Industry Collaboration:

☐ Partner with industry peers to develop shared blockchain frameworks and standards.

☐ Join consortia like Hyperledger or Ethereum Enterprise Alliance to gain insights and support.

2. Academic Partnerships:

☐ Collaborate with universities to conduct research and pilot innovative blockchain solutions.

☐ Offer internships or projects for students to contribute to blockchain initiatives.

Factor: Performance Metrics and Continuous Improvement

1. Key Metrics:

☐ Define clear KPIs such as transaction speed, cost savings, and user adoption rates.

☐ Use these metrics to evaluate blockchain’s impact and inform future decisions.

2. Feedback Loops:

☐ Establish mechanisms for gathering feedback from users and stakeholders.

☐ Regularly update blockchain strategies based on feedback and performance data.

Factor: Risk Management

1. Risk Identification:

☐ Identify potential risks such as technology obsolescence or vendor lock-in.

☐ Conduct scenario planning for unexpected challenges.

2. Mitigation Strategies:

☐ Develop contingency plans for system downtime or data breaches.

☐ Use smart contracts to automate compliance and reduce operational risks.

Factor: Organizational Culture

1. Innovation Mindset:

☐ Foster a culture that embraces innovation and is open to experimenting with new technologies.

☐ Recognize and reward teams or individuals driving blockchain initiatives.

2. Communication:

☐ Maintain open channels of communication to address misconceptions about blockchain.

☐ Share regular updates on project progress and success stories to build enthusiasm.

This expanded checklist ensures comprehensive planning, adoption, and scaling of blockchain technology while addressing key organizational priorities and potential challenges.

Factor: Strategic Planning

☐ Define Objectives: Clearly outline the purpose and goals of adopting blockchain technology (e.g., enhancing transparency, improving efficiency, reducing costs).

☐ Conduct a Feasibility Study: Assess the technical, financial, and operational feasibility of integrating blockchain into the organization’s processes.

☐ Develop a Roadmap: Establish a step-by-step strategy for blockchain implementation, including short-term and long-term goals.

☐ Identify Use Cases: Focus on areas where blockchain can provide the most value (e.g., supply chain management, financial transactions, identity verification).

Factor: Technological Readiness

☐ Assess IT Infrastructure: Evaluate the current IT systems to determine if they can support blockchain integration or require upgrades.

☐ Select a Blockchain Platform: Choose a blockchain technology (e.g., Ethereum, Hyperledger, Corda) based on the organization’s specific needs.

☐ Ensure Scalability: Verify that the chosen blockchain solution can handle the organization’s transaction volumes as they grow.

☐ Implement Middleware: Develop or adopt middleware to enable seamless integration between blockchain and existing systems.

Factor: Security and Privacy

☐ Conduct Risk Assessments: Identify potential security vulnerabilities in blockchain implementation and address them proactively.

☐ Implement Encryption Standards: Utilize advanced encryption protocols to safeguard sensitive organizational data.

☐ Ensure Data Privacy Compliance: Align blockchain implementation with relevant data privacy regulations (e.g., GDPR, HIPAA).

☐ Set Access Controls: Establish secure identity and access management policies to regulate who can view or modify blockchain data.

Factor: Regulatory Compliance

☐ Understand Legal Frameworks: Research applicable local, national, and international laws governing blockchain use in the organization’s sector.

☐ Collaborate with Regulators: Work with legal and regulatory bodies to ensure compliance and receive guidance on best practices.

☐ Develop Policies: Create internal policies to align blockchain usage with regulatory requirements, such as anti-money laundering (AML) and know-your-customer (KYC) standards.

☐ Monitor Regulatory Updates: Stay informed about changes in regulations that may impact blockchain operations.

Factor: Financial Planning

☐ Allocate Budget: Dedicate funds for blockchain implementation, including infrastructure, training, and ongoing maintenance.

☐ Explore Funding Opportunities: Seek grants, partnerships, or external funding to support blockchain initiatives.

☐ Conduct Cost-Benefit Analysis: Evaluate the financial implications of blockchain adoption to ensure a positive return on investment (ROI).

☐ Plan for Operational Costs: Include ongoing expenses such as network fees, software updates, and technical support in financial plans.

Factor: Organizational Support

☐ Gain Leadership Endorsement: Secure buy-in from senior management to champion blockchain initiatives.

☐ Create a Blockchain Task Force: Assemble a dedicated team to oversee blockchain implementation and operations.

☐ Engage Stakeholders: Communicate with internal and external stakeholders to align their expectations with blockchain goals.

☐ Foster Interdepartmental Collaboration: Encourage cooperation between departments to integrate blockchain seamlessly into organizational workflows.

Factor: Staff Training and Capacity Building

☐ Conduct Awareness Sessions: Educate employees on the basics of blockchain technology and its potential benefits.

☐ Provide Technical Training: Train IT teams to manage and maintain blockchain systems effectively.

☐ Develop User Guidelines: Create clear instructions for staff interacting with blockchain systems.

☐ Encourage Continuous Learning: Offer ongoing training programs to keep employees updated on blockchain advancements.

Factor: Pilot Projects

☐ Identify Pilot Areas: Choose low-risk, high-impact areas for initial blockchain implementation.

☐ Test Feasibility: Run pilot projects to evaluate blockchain’s practicality and benefits in specific organizational processes.

☐ Collect Feedback: Gather insights from pilot users to identify areas for improvement.

☐ Scale Based on Success: Expand blockchain usage to other areas based on the outcomes of pilot projects.

Factor: Collaboration and Partnerships

☐ Partner with Experts: Collaborate with blockchain experts, consultants, or technology providers for guidance and technical support.

☐ Establish Industry Alliances: Join industry consortia or working groups to stay informed about blockchain trends and best practices.

☐ Engage with Academic Institutions: Work with universities and research institutions to explore innovative blockchain applications.

☐ Foster Public-Private Partnerships: Collaborate with government agencies or private-sector partners to share resources and expertise.

Factor: Monitoring and Evaluation

☐ Define Key Performance Indicators (KPIs): Identify measurable metrics to assess the success of blockchain initiatives (e.g., transaction speed, cost savings, user satisfaction).

☐ Conduct Regular Audits: Periodically review blockchain operations to ensure alignment with organizational goals and compliance requirements.

☐ Use Real-Time Analytics: Leverage blockchain analytics tools to monitor system performance and identify areas for optimization.

☐ Iterate Based on Insights: Use evaluation results to refine and improve blockchain strategies.

Factor: Risk Management

☐ Develop a Risk Management Plan: Identify potential risks (e.g., data breaches, system failures) and outline mitigation strategies.

☐ Implement Backup Systems: Ensure data is backed up regularly and can be restored quickly in case of failure.

☐ Prepare Contingency Plans: Develop response strategies for unforeseen challenges in blockchain implementation.

☐ Address Change Management Risks: Create strategies to manage resistance and ensure smooth transitions during blockchain adoption.

Factor: Sustainability

☐ Assess Energy Efficiency: Choose blockchain solutions that minimize environmental impact (e.g., proof-of-stake consensus mechanisms).

☐ Promote Long-Term Viability: Develop a sustainable model for blockchain operations, including financial and operational aspects.

☐ Leverage Green Technologies: Explore renewable energy sources to power blockchain networks and reduce carbon footprint.

☐ Align with Corporate Social Responsibility (CSR): Integrate blockchain initiatives with the organization’s CSR goals to maximize societal benefits.

Factor: Innovation and Scalability

☐ Encourage Experimentation: Foster a culture of innovation by encouraging teams to explore creative blockchain applications.

☐ Plan for Future Growth: Ensure blockchain systems can scale as the organization expands.

☐ Stay Updated on Trends: Monitor advancements in blockchain technology to identify opportunities for innovation.

☐ Incorporate Feedback Loops: Use user feedback to drive continuous improvement and innovation in blockchain solutions.
